# New insights in IBS-like disorders: Pandora's box has been opened; a review

**Published:** 2017

**Authors:** Raffaele Borghini, Giuseppe Donato, Domenico Alvaro, Antonio Picarelli

**Affiliations:** 1 *Department of Internal Medicine and Medical Specialties, Sapienza University, Rome, Italy*; 2 *Department of Clinical Medicine, Sapienza University, Rome, Italy *; 3 *Department of Medico-Surgical Sciences and Biotechnologies, Sapienza University, Rome, Italy*

**Keywords:** Irritable bowel syndrome (IBS), Celiac disease, Non-celiac gluten sensitivity (NCGS), Lactose intolerance, Nickel, ATIs, SIBO

## Abstract

The most complained gastrointestinal symptoms are chronic diarrhea, bloating and abdominal pain. Once malignancies and inflammatory bowel diseases are excluded, irritable bowel syndrome (IBS) and the so called “IBS-like disorders” should be taken into account. The relationship between IBS as defined by Rome IV criteria and these clinical conditions is sometimes obscure, since many IBS patients identify food as a possible trigger for their symptoms. Here, we discuss IBS and the most common IBS-like disorders (celiac disease, non-celiac gluten sensitivity, fermentable oligosaccharides, disaccharides, monosaccharides and polyols (FODMAPs), lactose intolerance, small intestinal bacterial overgrowth (SIBO), α-amylase/trypsin inhibitor (ATIs), nickel allergic contact mucositis), focusing on epidemiologic, clinical, diagnostic and therapeutic aspects. Given the lack of specificity of symptoms, clinical investigation will be facilitated by awareness of these disorders as well as new specific diagnostic tools.

## Introduction

 Nowadays, when a patient is referred to the gastroenterologist, the reason is often linked to chronic diarrhea, bloating and abdominal pain. As is known, this nonspecific clinical picture can be observed in many conditions, including gastrointestinal malignancies, inflammatory bowel diseases (IBD) and the well-known irritable bowel syndrome (IBS). In addition, more recently, other pathologic conditions, such as food allergies and other adverse reactions to foods are increasingly gaining visibility in the scenery of what can be called “IBS-like disorders”, since their clinical appearance may overlap with that of IBS: in fact, it is well recognized that there are probably many conditions with different pathogenetic mechanisms that are currently labeled under one large umbrella as "IBS".

The relationship between IBS as defined by Rome IV criteria (1) and these clinical conditions is still somewhat controversial (2). Under certain ambiguous circumstances, an exclusive and pure diagnosis of IBS cannot be achieved because of food-dependent symptoms: in fact, up to 80% of IBS patients identify food as a possible trigger for their symptoms, so they increasingly ask for dietary and behavioral counseling (3). To avoid misconceptions, there are no known exclusion criteria for IBS; thus, the Rome IV criteria seem unable to exclude an underlying possible IBS-like disorder. We will discuss IBS and the most common IBS-like disorders, focusing on epidemiologic, clinical, diagnostic and therapeutic aspects. 

**Irritable Bowel Syndrome**

IBS is a functional bowel disorder and one of the most commonly diagnosed gastrointestinal illnesses. It is a symptom-based condition characterized by abdominal pain or discomfort, with altered bowel habits, in the absence of any other disease to cause these sorts of symptoms. 

Its estimated prevalence is 10%–20% (4), although marked variation may exist based on geographical location; for example, its prevalence is 21% in South America versus 7% in Southeast Asia (5). It is nearly twice more common in women than men (6). In the United States, patients are equally distributed among IBS with diarrhea (IBS-D), IBS with constipation (IBS-C), and IBS with a mixed bowel pattern (IBS-M), whereas in Europe, studies have found either IBS-C (45.9%) or IBS-D (50%) as the main pattern group (7, 8). 

The heterogeneous pathogenesis of IBS seems to imply alterations in motility, visceral sensation, brain–gut interactions, microbiome, bile acid metabolism, and intestinal permeability. Moreover, an immune activation is probably involved in a sort of low-grade inflammation. Actually, colonic mucosal biopsies of about two-thirds of patients with IBS show a dense mast cell infiltrate which releases many mediators, such as serine proteases, probably responsible for neuronal hyperexcitability and IBS symptoms. Moreover, food components and antigens are believed to pass through a leaky epithelial barrier, leading to mast cell infiltration and activation, thereby leading to IBS symptoms (9). Since mast cells can be activated by allergy-like mechanisms and standard skin-prick tests have poor sensitivity and specificity, an immune response to food in IBS has been even tested in the past with a non-conventional approach, a sort of ‘mucosal prick test’ known as colonoscopic allergen provocation (COLAP) test, which involves colonoscopy-guided submucosal injection to unravel food hypersensitivity (10). Seventy-seven percent of a population with gut symptoms thought possibly related to food hypersensitivity had a positive COLAP test, which was consistently negative in the few control subjects. In addition, confocal laser endomicroscopy is a more refined technique which consists of submucosal injection of food antigens causing increased infiltration with intraepithelial lymphocytes (IELs), formation of epithelial leaks/gaps and widening of intervillous spaces in more than half of IBS cases, and not in a small group of controls (11). However, more studies are needed to confirm the diagnostic relevance of these invasive tests.

Treatment strategies for IBS may include both nonpharmacologic and pharmacologic approaches. Lifestyle modifications improving exercise, sleep, diet, and stress are sometimes recommended. On the other hand, IBS-D patients can be treated with synthetic peripheral μ-opioid receptor agonist loperamide, antispasmodic agents, antidepressants, serotonin 5-HT3 antagonists, and the gut-specific antibiotic rifaximin, whereas the usefulness of probiotics is still under consideration. For IBS-C patients, therapeutic strategies may include dietary fiber, laxatives, and prosecretory agents lubiprostone and linaclotide (12). Despite significant research efforts, unfortunately symptom-targeted treatments currently available for IBS do not guarantee definitive and lasting solutions, as they can offer therapeutic gains of about 20% over placebo and only half or fewer patients actually gain benefit (6, 7). 

Thus, an accurate differential diagnosis is necessary in order to exclude common gastrointestinal disorders, such as CD, food allergies and, more specifically, adverse reactions to foods (13). These conditions are becoming more and more common among IBS patients, despite lack of epidemiological and etiopathogenetic knowledge, as well as sensitive and specific diagnostic tools. 

**Celiac disease**

CD is a chronic inflammatory bowel disease triggered by ingestion of gluten which affects genetically susceptible individuals who are positive for HLA DQ2 (90–95%) and DQ8 (5–10%). Its prevalence is about 1% in the general population. The disease primarily involves the intestinal tract and usually presents with diarrhea, bloating, abdominal pain, and weight loss as a consequence of malabsorption (14). On the other hand, many extra-intestinal manifestations may also be found in CD patients to make diagnosis even more difficult, such as iron deficiency anemia, osteopenia and osteoporosis, arthritis, dermatitis herpetiformis, eczema and psoriasis, and even gluten ataxia and autism. Hepatic, cardiovascular, pulmonary, pancreatic, reproductive and dental manifestations may also be observed. Most of them share an inflammatory and/or autoimmune etiology (15). 

CD diagnosis is based on the finding of villous atrophy on histologic examination of duodenal biopsies during gluten-containing diet (16), supported by specific and sensitive serological IgA and/or IgG anti-tissue transglutaminase (anti-tTG) and anti-endomysial antibodies (EMA) (17, 18). Moreover, the in vitro gluten challenge test for EMA and anti-tTG using duodenal biopsy supernatant revealed to be a useful part of the diagnostic workup of CD, especially in the most controversial cases (19). The only treatment currently available for CD is a life-long gluten-free diet (GFD) (14). 

**Non-celiac Gluten Sensitivity**

As already mentioned and stated in the current literature, wheat gluten could cause a range of adverse gastrointestinal and extra-intestinal disorders, including wheat allergy and CD, with prevalence rates of about 0.1% and 1%, respectively. Their diagnosis has been improved by increased awareness, as well as sensitive and specific tools, and the only treatment available is GFD (14). However, there are other adverse reactions to wheat and gluten that are more difficult to diagnose, since their immunopathologic process is not still clear. In particular, non-celiac gluten sensitivity (NCGS) is emerging as a new clinical entity lacking specific diagnostic biomarkers and it has been reported to occur in 6-10% of the population, although up to 75% of these patients carry HLA-DQ2 and/or HLA-DQ8 genes (13). NCGS patients may complain CD-like or IBS-like symptoms (e.g. diarrhea, abdominal pain, bloating) which recede with GFD, as well. So far, only one diagnostic protocol has been recommended for confirmation of NCGS (20), according to which a clinical evaluation should be performed using a self-administered questionnaire. Patients identify symptoms that are quantitatively assessed according to a Numerical Rating Scale (ranging from 1 to 10) and a double-blind placebo-controlled challenge with gluten 8 g/day is performed. This method has been proposed to facilitate possible comparisons among different international studies, but it requires strict compliance rules for patients and a very long time to reach a diagnosis. Recently, Picarelli et al. (13) developed an oral mucosa patch test for gluten (GOMPT) that may be considered a reliable and rapid tool to confirm the diagnosis of NCGS, with 100% specificity and 75% sensitivity. In detail, since oral and intestinal mucosa have the same embryonic origin, the upper lip mucosa is used as application site of this device to evaluate local and general reactions triggered by direct contact with gluten. Two hours after the administration of GOMPT, NCGS patients usually indicate mucosal hyperemia, edema, blisters and burning at the test site. Within 48 hours, they may report general reactions such as diarrhea, abdominal swelling, abdominal pain, foggy mind, fatigue, itching, headache and arthralgia. GOMPT seems to be a useful tool for NCGS diagnosis, although further investigations are needed to overcome limits due to the small population studied so far in a single diagnostic center. Moreover, its sensitivity could be improved by using specific laser Doppler perfusion imaging (LDPI), as already proposed more recently (21). 

**FODMAPs**

In recent years, fermentable oligosaccharides, disaccharides, monosaccharides and polyols (FODMAPs) have been increasingly recognized as a possible trigger of symptoms compatible with a diagnosis of IBS, and a diet low in FODMAPs has been even suggested as a strategy to improve symptoms in patients with IBS, irrespective of the underlying cause. FODMAPs are poorly absorbed, short-chain carbohydrates comprised of small osmotically active molecules that can lead to excessive fluid and gas accumulation, resulting in bloating, abdominal pain, and distention. FODMAPs are present in many foods containing lactose, fructose in excess of glucose, fructans, galacto-oligosaccharides, and polyols (sorbitol, mannitol, xylitol, and maltitol). FODMAPs are poorly absorbed mainly because of the absence of luminal enzymes capable of hydrolyzing the glycosidic bonds existing in carbohydrates, the absence or low activity of brush border enzymes (e.g. lactase), or the presence of low-capacity epithelial transporters (fructose, glucose transporter 2 [GLUT-2], and glucose transporter 5 [GLUT-5]). The fermentation rate is determined by the chain length of the carbohydrate. Moreover, polyols are too large for simple diffusion (22).

Although with small sample sizes, many studies and randomized controlled trials have reported a good control of IBS symptoms after a diet low in FODMAPs, with improvement in overall gastrointestinal symptoms in as high as 68-86% of IBS patients (23, 24).

However, there is also evidence that intensive restriction of FODMAPs could potentially have long-term negative consequences, both from a nutritional point of view, and the impact on the intestinal microbiota. Thus, identifying the most offending FODMAPs in specific patients could mitigate dietary restrictions (25).

**Lactose Intolerance**

Lactose, a disaccharide consisting of galactose bound to glucose, can cause intolerance related to primary or secondary lactase deficiency. The most frequent cause of lactose malabsorption is lactase non-persistence, whereas acquired conditions include small intestinal bowel overgrowth (SIBO), infectious enteritis (e.g. giardiasis), or mucosal alterations secondary to CD, inflammatory bowel disease (IBD), drugs, surgery, radiations, or other conditions that imply the reduction of absorptive capacity or downregulation of lactase expression in the small intestine.

Lactase activity in Caucasian individuals is mainly encoded by the genetic polymorphism C/T-13910 variant, and a second variant, G/A-22018, 8 kb telomeric to C/T-13910, is also associated with the trait (26). Lactase activity progressively declines during development, reaching a stable low level at the age of 10 years. The prevalence of lactase persistence is characterized by geographic variation: for example, it is extremely high in northern European populations, reaching 90% in Scandinavia or Holland. On the contrary, it progressively decreases in southern Europe/Middle East (50% in Spain, Italy and pastoralist Arab populations), and India (20-40%) and reaches its lowest percentages in Africa (5%–20%) and Asia (1% in China). Tutsi and Fulani, pastoralist populations from Africa, are exceptions, with a prevalence of lactase persistence of respectively 90% and 50% (27). 

Lactose intolerance is mainly characterized by abdominal pain and swelling, diarrhea and flatus and it is induced by lactose in milk and dairy products. These symptoms seem to depend on the dose of lactose ingested (they are extremely rare after ingestion of <10 g lactose), as well as the intestinal flora.

In a retrospective case review, an interesting intersection was observed between lactose intolerance and IBS, reporting an improvement of abdominal discomfort, with lactose restriction in up to 85% of IBS patients with lactose malabsorption (28); however, prospective studies show that lactose restriction alone is not sufficient for effective symptom relief in functional GI disease (27).

Nowadays, many diagnostic tests are available for this condition, including H2-breath test, lactose tolerance test, genetic test of C/T-13910 and G/A-22018 polymorphisms and assessment of lactase activity at jejunal brush border. Each test is characterized by different principle, availability, sensitivity, specificity and cost. Treatment of lactose intolerance should imply reduction of lactose intake rather than exclusion, considering tolerability and consequent nutritional deficiencies, especially for menopausal women. Enzyme replacement therapy for primary adult lactase deficiency is also available, improving diet variability and, in general, quality of life (29).

**Small Intestinal Bacterial Overgrowth**

The presence of SIBO has been documented in patients with IBS and, although this issue is causing a heated controversy, a reduction in SIBO seems to correlate with improvement of IBS symptom in some clinical trials. SIBO should always be considered as a differential diagnosis in patients with refractory functional diseases and IBS (30). 

SIBO is a condition in which an alteration occurs in both quantity and quality of gut microbiota in the small intestine, involving beneficial and pathogenic species. It is usually defined as >105 aerobic and anaerobic coliforms per mL, although some studies have considered a lower concentration (>103 colony-forming units/mL). 

The prevalence of SIBO in the general population is unknown, ranging from 0 to 20% in apparently healthy controls, with significant changes in apparently associated disorders. Specifically, the reported prevalence of SIBO in IBS patients is generally high, ranging from 4 to 64% (84% in some cases) and mainly involving patients with IBS-D. These great variations in epidemiologic data surely pertain to lack of a ‘gold’ standard for SIBO definition and diagnosis (31, 32).

SIBO diagnosis is conventionally based on jejunal aspirate culture findings, but sample collection is invasive and isolated distal SIBO often remains undiagnosed because of limited access. Other limitations can be potential sample contaminations or possible false negative results (31). For these reasons, hydrogen breath tests using glucose or lactulose have been proposed and widely used as a noninvasive diagnostic tools for SIBO, despite their uncertain accuracy. Specifically, glucose hydrogen breath test (GHBT) showed fewer false-positive results compared to lactulose hydrogen breath test (LHBT), making GHBT the most widely used technique for diagnosing SIBO (33). Some authors have even demonstrated a moderate agreement (65.5–77.7%) between duodenal culture and GHBT depending on whether 103 or 105 colony-forming units/mL was used as cutoff. However, further research is required to delineate the diagnostic criteria for SIBO more clearly (34, 35).

The possible role of gut microbiota in the pathophysiology of IBS and IBS-like disorders such as SIBO is supported by the efficacy of some probiotics and nonsystemic antibiotics in their treatment. Rifaximin, a poorly absorbed rifamycin derivative with a high level of solubility in the small intestine, can have bactericidal activity resetting microbial diversity. Moreover, it may also decrease host proinflammatory responses to bacterial products in patients with IBS or SIBO. All these conditions may lead to a decrease in bacterial fermentation and reduction in clinical symptoms, although the precise mechanisms of action of short-term treatment with rifaximin are not completely clear (31). 

**α-Amylase/ Trypsin Inhibitors**

The recent breeding of highly pest-resistant wheat has led to a drastic increase of nongluten α-amylase/trypsin inhibitor (ATIs), which are primary resistance molecules contained in wheat or related cereals to fend off pests and parasites. In details, wheat ATIs are a family of up to 17 water soluble globulins of about 15 kD molecular weight and represent between 2-4% of total wheat protein (as compared to ~80-90% for gluten) (36).

Recent studies have focused on the potential immunological role of ATIs in developing intestinal and extra-intestinal manifestations in CD or IBS-like patients (9). After in vitro and in vivo experiments, some members of the ATI family have been identified as strong inducers of innate immune responses in human and murine macrophages, monocytes, and dendritic cells. Specifically, these cereal ATIs proved to engage the toll-like receptor (TLR)4–MD2–CD14 complex, leading to up-regulation of maturation markers and eliciting release of proinflammatory cytokines (e.g. TNFα and IL8) in cells from CD and non-celiac patients, as well as in CD patients’ biopsy cultures. Moreover, mice deficient in TLR4 or TLR4 signaling were shown to be protected against intestinal and systemic immune responses upon oral challenge with ATIs (37).

These findings may open new possible scenarios for the etiopathogenesis and treatment not only of CD, but also of IBS-like gastrointestinal disorders which have been so far unknown or vastly underestimated, such as NCGS. ATIs may even have implications for the course of IBD, since a large number of IBD patients attempting a GFD report some signs of improvement in gastrointestinal symptoms (37). Moreover, cereal ATIs revealed to be possible contributors to inflammation and immune reactions in non-intestinal immune disorders (38).

**Nickel Allergic Contact Mucositis**

Dietary nickel (Ni) was recently revealed as a possible causative factor in patients with IBS-like symptoms. Even a large percentage of patients with suspected NCGS can actually have an allergy to Ni (39), or, better, “adverse reactions to Ni-containing foods”.

**Figure 1:**
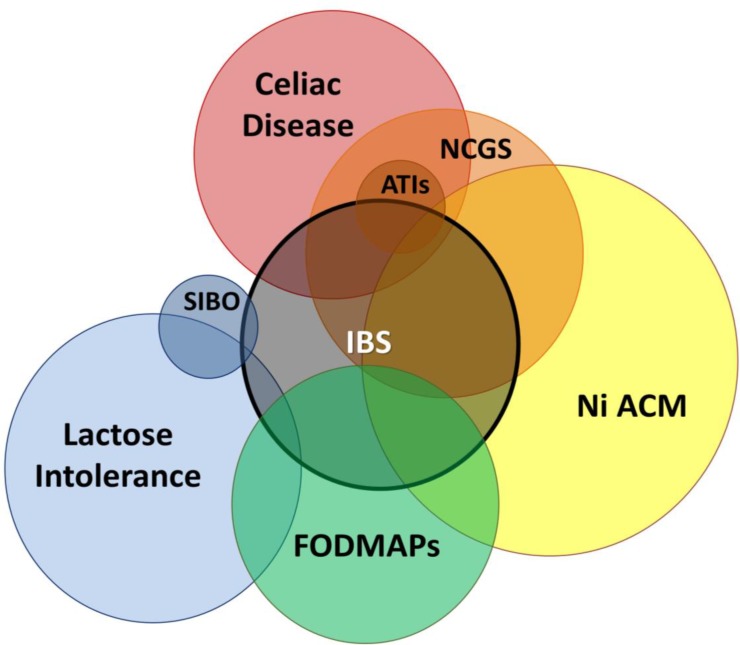
Clinical overlap between IBS and IBS-like disorders. IBS, Irritable Bowel Syndrome; FODMAPs, Fermentable Oligosaccharides, Disaccharides, Monosaccharides and Polyols; SIBO, Small Intestinal Bacterial Overgrowth; NCGS, Nonceliac Gluten Sensitivity; ATIs, α-Amylase/Trypsin Inhibitors; Ni ACM, Nickel Allergic Contact Mucositis

Ni is a ubiquitous element in nature, which can be ingested, come into contact with the skin or be inhaled. According to the European Surveillance System of Contact Allergy (ESSCA), the prevalence of epicutaneous patch test positive to Ni is about 30% in Europe. The gastrointestinal tract can represent a route of exposure to this element, inducing systemic Ni allergy syndrome (SNAS), which can be characterized not only by contact dermatitis but also by extra-cutaneous signs and symptoms. Apart from respiratory and neurological manifestations, Ni-related symptoms in Ni-sensitive patients are mainly gastrointestinal. Ni-rich foods include tomato, cocoa, beans, mushrooms, broad-leafed vegetables, whole-wheat flour, corn, onion, garlic, shellfish, nuts, and foods contained in aluminum boxes (21).

As a precursor of GOMPT, a Ni oral mucosa patch test (Ni omPT) has been proposed and revealed useful for the diagnosis of Ni allergic contact mucositis (ACM), whose prevalence may even be higher than 30% (40). It consists of a 5-mm filter paper disk saturated with a 5% solution of Ni sulfate in Vaseline (0.4 mg Ni-sulfate/8 mg Vaseline) applied on the lower lip mucosa. After 2 hours of exposure, it induces specific local alterations on labial mucosa of Ni-sensitive patients, such as edema and hyperemia, probably related to a TLR4-dependent innate immune response. Aphthous/vesicular lesions may appear even after 24–48 hours, identified as a type IV hypersensitivity reaction (41). The effectiveness of Ni OmPT has been proven, but to overcome limits due to the operator-dependence of the method, it has been combined with LDPI to support omPT in Ni ACM diagnosis. This technology can calculate precisely the mean mucosal perfusion at the test site after omPT. Moreover, it is suitable for symptomatic Ni-sensitive patients without aphthous stomatitis after 24-48 hours from omPT and that could pose a risk to diagnosis (21).

In many studies, a diet low in Ni is revealed to be effective, inexpensive and without significant side effects, apart from high risk of constipation due to poor fiber intake. 

Regarding adverse reactions to Ni-containing foods, many possible treatment options have been proposed in the last 50 years in the scientific literature.

Disulfiram, a Ni chelator, has been proposed as possible treatment for Ni-sensitivity, but it has potential hepatotoxicity (42). Intravenous injection of ethylene diamine tetraacetic acid (EDTA) has also been proposed in the past as chelation therapy, with the purpose of eliminating from the body heavy metals, chemical toxins, mineral deposits and fatty plaques cancer. Kidneys are the only organs commonly affected by EDTA toxicity, although EDTA is approximately only one third as toxic as aspirin (43). Only few cases of bone marrow suppression, gastrointestinal and musculoskeletal symptoms, as well as fatal episodes have been reported during EDTA therapies. However, more studies about metal balance and drug interactions are required to delineate the risks and benefits of EDTA long term treatment. For example, prolonged use of EDTA may cause depletion of essential metals such as iron, zinc, cobalt, manganese and copper (44). 

Natural clinoptilolite zeolite can be used as “ion exchanger” for removal of heavy metals - and Ni in particular - and it has already been used for treatment of environmental pollution because of its desirable characteristics of high ion exchange selectivity and resistance to different media (45). However, the scientific community still lacks clinical data about clinoptilolite oral intake as possible treatment of adverse reactions to Ni-rich foods. 

**Table 1:** IBS and IBS-like disorders: main features

IBS-like disorders	prevalence	cause/trigger	diagnostic test	treatment
IBS	10-20%	alteration in motility	Rome IV criteria	lifestyle modifications
		alteration of visceral sensation		loperamide
		brain–gut interactions		antispasmodic agents
		microbiome		serotonin 5-HT3 antagonists
		bile acid metabolism		rifaximin
		intestinal permeability		probiotics
		immune activation		fiber, laxatives
		leaky epithelial barrier		lubiprostone, linaclotide
		others?		others?
Celiac Disease	1%	gluten	IgA/IgG EMA and anti-tTG (in serum)	gluten-free diet
			histology (biopsy from bulb/II portion of duodenum)	
			IgA/IgG EMA and anti-tTG (in duodenal organ culture supernatant)	
			HLA DQ2/DQ8 (high negative predictive value)	
NCGS	6-10%	gluten	Salerno Experts' Criteria	gluten-free diet
			GOMPT (+ LDPI)	
FODMAPs	unknown	fermentable oligosaccharides, disaccharides, monosaccharides and polyols	unknown	low-FODMAP diet
Lactose Intolerance	geographically variable (50% in southern Europe/Middle East)	primary or secondary lactase deficiency	H2-breath test	low-lactose diet
			genetic test (C/T-13910 and G/A-22018)	enzyme replacement therapy (lactase)
SIBO	4-68%	alteration of gut microbiota in the small intestine	GHBT	probiotics
			LHBT	antibiotics
			jejunal aspirate culture	rifaximin
ATIs	unknown	nongluten α-amylase/trypsin inhibitor in wheat or cereals to fend off parasites	unknown	wheat/gluten free diet (?)
Ni ACM	>30%	dietary Ni	Ni omPT (+ LDPI)	low-Ni diet
				ascorbic acid
				iron supplements
				chelating agent (clinoptilolite zeolite) (?)

Alternative ways to decrease dietary Ni absorption and promote its fecal excretion can be also recommended, such as iron supplementation or addition of iron-rich foods to the diet. Since Ni and iron share and compete for absorptive pathways, it can be expected that iron intake can limit Ni absorption, while individuals with low iron status may be at risk for increased Ni absorption (46). Moreover, ascorbic acid supplementation can be useful, as it has been shown to reduce the rise in plasma Ni concentration (42). As an antioxidant agent, it could also play an important role in protecting proteins against Ni-induced oxidative stress and in abating the genotoxic hazards of Ni (47).

## Discussion

IBS diagnosis is based on Rome IV criteria; however, since IBS patients often correlate their symptoms with the ingestion of specific foods, a pure and exclusive diagnosis of IBS cannot be reached. Thus, a clinical overlap between IBS and other IBS-like disorders can be suggested (2). As summarized in Figure 1, each of the above mentioned IBS-like conditions has clinical features (chronic diarrhea, bloating, abdominal pain) which may overlap with IBS itself. On the other hand, sometimes incomplete knowledge of the etiopathogenesis of these clinical conditions, lack of data on their real epidemiology, as well as the absence of a gold standard for their diagnosis, make the overall picture difficult to understand (Table 1).

As far as symptoms are concerned, CD and IBS share many similarities. On the basis of this clinical overlap and although it is inevitable that CD and IBS will sometimes occur in the same individual, a misdiagnosis of CD and an over-diagnosis of IBS may be also common. It is reported that about 10% of CD patients receive an incorrect diagnosis of IBS prior to receiving their diagnosis of CD and spend many years being treated as IBS patients. To make things more complicated, both patients and physicians may also be deceived by the role of wheat products: in CD, gluten contained in wheat is the trigger for autoimmunity, while in the case of IBS, the clinical effects could be due to long sugar polymer fructose found in wheat. Following guidelines to test serologically for CD should minimize this problem (48, 49).

Focusing further on the relationship between IBS and the ingestion of gluten, Shahbazkhani B. et al. recently published the results of a double-blind randomized placebo-controlled trial and confirmed that, even after the exclusion of CD, a statistically significant improvement in symptoms can be obtained in a high percentage of IBS patients with GFD. Thus, identifying NCGS patients in the spectrum of IBS may be a priority (50). In addition, NCGS as well as lactose intolerance and Ni ACM may be considered among the most frequent IBS-like conditions and their diagnosis may be supported by tools already discussed (13, 26).

FODMAPs have been shown to share clinical characteristics and trigger foods with both lactose intolerance and Ni ACM. Specifically, many foods rich in FODMAPs are also high in lactose. Given the high prevalence of lactose intolerance, it is not surprising that a diet low in FODMAPs may reduce or even resolve gastrointestinal and extra-intestinal symptoms. The same thing can be true for foods rich in Ni, very numerous in the FODMAPs family, such as pears, cabbage, garlic, onion and legumes. Another important intersection exists between FODMAPs and NCGS, or even better between Ni ACM and NCGS: upon closer analysis, symptoms of suspected NCGS patients are actually triggered by associated Ni-rich ingredients or condiments (e.g. yeast or tomato), and not by gluten itself. As a consequence, foods such as bread, pasta with tomato sauce, pizza and bakery products turn into real traps for Ni-sensitive and/or lactose intolerant patients, in defiance of the Mediterranean diet, recently declared part of the UNESCO's Intangible Heritage List. 

Other important considerations can be made during serological and histological remission in CD patients following a strict GFD: at this stage, it is quite common to observe an apparently unjustified recurrence (or an ex novo outbreak) of both intestinal and extra-intestinal symptoms. In these cases, a latent or overlapped Ni ACM may be exacerbated by chronic ingestion of gluten-free products rich in Ni, such as corn pasta. This is often the keystone of such frequent clinical events, which resolve once an adequate low-Ni diet is started.

As reported above, both lactose intolerance and Ni ACM can be supported by therapeutic strategies, such as enzyme replacement therapy, antioxidants or chelating treatments, as well as use of competitors. It results in avoiding nutritional deficiencies and improving variability in the diet itself. 

Yet to be explored are ATIs, which, together with Ni ACM, seem to be another possible keystone in the interpretation of symptoms in suspected NCGS. 

## Conclusion

In conclusion, once major organic gastrointestinal disorders are excluded, specialists should always take into account all the possible diagnoses labeled under the large umbrella of "IBS", given the lack of specificity of symptoms. In this regard, the diagnostic investigation will be facilitated by both the awareness of these disorders and a careful analysis of food record diary and alimentary anamnesis.

## Conflict of interest

The authors declare that they have no conflict of interest. 

## References

[1] Drossman DA (2016). Functional gastrointestinal disorders: history, pathophysiology, clinical features and Rome IV. Gastroenterology.

[2] Makharia A, Catassi C, Makharia GK (2015). The Overlap between Irritable Bowel Syndrome and Non-Celiac Gluten Sensitivity: A Clinical Dilemma. Nutrients.

[3] Chey WD (2016). Food: The Main Course to Wellness and Illness in Patients With Irritable Bowel Syndrome. Am J Gastroenterol.

[4] Longstreth GF, Thompson WG, Chey WD, Houghton LA, Mearin F, Spiller RC (2006). Functional bowel disorders. Gastroenterology.

[5] Staudacher HM, Irving PM, Lomer MC, Whelan K (2014). Mechanisms and efficacy of dietary FODMAP restriction in IBS. Nat Rev Gastroenterol Hepatol.

[6] Chey WD, Kurlander J, Eswaran S (2015). Irritable bowel syndrome: a clinical review. JAMA.

[7] Ford AC, Moayyedi P, Lacy BE, Lembo AJ, Saito YA, Schiller LR (2014). American College of Gastroenterology monograph on the management of irritable bowel syndrome and chronic idiopathic constipation. Am J Gastroenterol.

[8] Guilera M, Balboa A, Mearin F (2005). Bowel habit subtypes and temporal patterns in irritable bowel syndrome: systematic review. Am J Gastroenterol.

[9] De Giorgio R, Volta U, Gibson PR (2016). Sensitivity to wheat, gluten and FODMAPs in IBS: facts or fiction?. Gut.

[10] Bischoff SC, Mayer J, Wedemeyer J, Meier PN, Zeck-Kapp G, Wedi B (1997). Colonoscopic allergen provocation (COLAP): a new diagnostic approach for gastrointestinal food allergy. Gut.

[11] Fritscher-Ravens A, Schuppan D, Ellrichmann M, Schoch S, Röcken C, Brasch J (2014). Confocal endomicroscopy shows food-associated changes in the intestinal mucosa of patients with irritable bowel syndrome. Gastroenterology.

[12] Lacy BE, Chey WD, Lembo AJ (2015). New and Emerging Treatment Options for Irritable Bowel Syndrome. Gastroenterol Hepatol (N Y).

[13] Picarelli A, Borghini R, Di Tola M, Marino M, Urciuoli C, Isonne C (2016). Intestinal, Systemic, and Oral Gluten-related Alterations in Patients With Nonceliac Gluten Sensitivity. J Clin Gastroenterol.

[14] Picarelli A, Borghini R, Isonne C, Di Tola M (2013). Reactivity to dietary gluten: new insights into differential diagnosis among gluten related gastrointestinal disorders. Pol Arch Med Wewn.

[15] Leffler DA, Green PH, Fasano A (2015). Extraintestinal manifestations of coeliac disease. Nat Rev Gastroenterol Hepatol.

[16] Picarelli A, Borghini R, Donato G, Di Tola M, Boccabella C, Isonne C (2014). Weaknesses of histological analysis in celiac disease diagnosis: new possible scenarios. Scand J Gastroenterol.

[17] Leffler DA, Schuppan D (2010). Update on serologic testing in celiac disease. Am J Gastroenterol.

[18] Rashid M, Lee J (2016). Serologic testing in celiac disease: Practical guide for clinicians. Can Fam Physician.

[19] Khalesi M, Jafari SA, Kiani M, Picarelli A, Borghini R, Sadeghi R (2016). In Vitro Gluten Challenge Test for Celiac Disease Diagnosis. J Pediatr Gastroenterol Nutr.

[20] Catassi C, Elli L, Bonaz B, Bouma G, Carroccio A, Castillejo G (2015). Diagnosis of Non-Celiac Gluten Sensitivity (NCGS): The Salerno Experts' Criteria. Nutrients.

[21] Borghini R, Puzzono M, Rosato E, Di Tola M, Marino M, Greco F (2016). Nickel-Related Intestinal Mucositis in IBS-Like Patients: Laser Doppler Perfusion Imaging and Oral Mucosa Patch Test in Use. Biol Trace Elem Res.

[22] Magge S, Lembo A (2012). Low-FODMAP Diet for Treatment of Irritable Bowel Syndrome. Gastroenterol Hepatol (N Y).

[23] Staudacher HM, Lomer MC, Anderson JL, Barrett JS, Muir JG, Irving PM (2012). Fermentable carbohydrate restriction reduces luminal bifidobacteria and gastrointestinal symptoms in patients with irritable bowel syndrome. J Nutr.

[24] Nanayakkara WS, Skidmore PM, O'Brien L, Wilkinson TJ, Gearry RB (2016). Efficacy of the low FODMAP diet for treating irritable bowel syndrome: the evidence to date. Clin Exp Gastroenterol.

[25] McIntosh K, Reed DE, Schneider T, Dang F, Keshteli AH, De Palma G (2016). FODMAPs alter symptoms and the metabolome of patients with IBS: a randomised controlled trial. Gut.

[26] Enattah NS, Sahi T, Savilahti E, Terwilliger JD, Peltonen L, Järvelä I (2002). Identification of a variant associated with adult-type hypolactasia. Nat Genet.

[27] Deng Y, Misselwitz B, Dai N, Fox M (2015). Lactose Intolerance in Adults: Biological Mechanism and Dietary Management. Nutrients.

[28] Böhmer CJ, Tuynman HA (2001). The effect of a lactose-restricted diet in patients with a positive lactose tolerance test, earlier diagnosed as irritable bowel syndrome: a 5-year follow-up study. Eur J Gastroenterol Hepatol.

[29] Misselwitz B, Pohl D, Frühauf H, Fried M, Vavricka SR, Fox M (2013). Lactose malabsorption and intolerance: pathogenesis, diagnosis and treatment. United European Gastroenterol J.

[30] Thompson JR (2016). Is irritable bowel syndrome an infectious disease?. World J Gastroenterol.

[31] Pimentel M (2016). Review article: potential mechanisms of action of rifaximin in the management of irritable bowel syndrome with diarrhoea. Aliment Pharmacol Ther.

[32] Quigley EM (2014). Small intestinal bacterial overgrowth: what it is and what it is not. Curr Opin Gastroenterol.

[33] Shimura S, Ishimura N, Mikami H, Okimoto E, Uno G, Tamagawa Y (2016). Small Intestinal Bacterial Overgrowth in Patients with Refractory Functional Gastrointestinal Disorders. J Neurogastroenterol Motil.

[34] Ghoshal UC, Srivastava D (2014). Irritable bowel syndrome and small intestinal bacterial overgrowth: meaningful association or unnecessary hype. World J Gastroenterol.

[35] Erdogan A, Rao SS, Gulley D, Jacobs C, Lee YY, Badger C (2015). Possible underestimation of SIBO in IBS patients: is lack of Glucose Breath Test standardization responsible?. Neurogastroenterol Motil.

[36] Schuppan D, Pickert G, Ashfaq-Khan M, Zevallos V (2015). Non-celiac wheat sensitivity: differential diagnosis, triggers and implications. Best Pract Res Clin Gastroenterol.

[37] Junker Y, Zeissig S, Kim SJ, Barisani D, Wieser H, Leffler DA (2012). Wheat amylase trypsin inhibitors drive intestinal inflammation via activation of toll-like receptor 4. J Exp Med.

[38] Herfarth HH, Martin CF, Sandler RS, Kappelman MD, Long MD (2014). Prevalence of a gluten-free diet and improvement of clinical symptoms in patients with inflammatory bowel diseases. Inflamm Bowel Dis.

[39] Volta U, Bardella MT, Calabrò A, Troncone R, Corazza GR (2014). Study Group for Non-Celiac Gluten Sensitivity An Italian prospective multicenter survey on patients suspected of having non-celiac gluten sensitivity. BMC Med.

[40] Picarelli A, Di Tola M, Vallecoccia A, Libanori V, Magrelli M, Carlesimo M (2011). Oral mucosa patch test: a new tool to recognize and study the adverse effects of dietary nickel exposure. Biol Trace Elem Res.

[41] Di Tola M, Marino M, Amodeo R, Tabacco F, Casale R, Portaro L (2014). Immunological characterization of the allergic contact mucositis related to the ingestion of nickel-rich foods. Immunobiology.

[42] Zirwas MJ, Molenda MA (2009). Dietary nickel as a cause of systemic contact dermatitis. J Clin Aesthet Dermatol.

[43] Foreman H (1963). Toxic side effects of ethylenedia minetetraacetic acid. J Chronic Dis.

[44] Born T, Kontoghiorghe CN, Spyrou A, Kolnagou A, Kontoghiorghes GJ (2013). EDTA chelation reappraisal following new clinical trials and regular use in millions of patients: review of preliminary findings and risk/benefit assessment. Toxicol Mech Methods.

[45] Oter O, Akcay H (2007). Use of natural clinoptilolite to improve water quality: sorption and selectivity studies of lead(II), copper(II), zinc(II), and nickel(II). Water Environ Res.

[46] Tallkvist J, Bowlus CL, Lönnerdal B (2003). Effect of iron treatment on nickel absorption and gene expression of the divalent metal transporter (DMT1) by human intestinal Caco-2 cells. Pharmacol Toxicol.

[47] Das KK, Buchner V (2007). Effect of nickel exposure on peripheral tissues: role of oxidative stress in toxicity and possible protection by ascorbic acid. Rev Environ Health.

[48] Al-Ajlan AS (2016). Screening of coeliac disease in undetected adults and patients diagnosed with irritable bowel syndrome in Riyadh, Saudi Arabia. Saudi J Biol Sci.

[49] Card TR, Siffledeen J, West J, Fleming KM (2013). An excess of prior irritable bowel syndrome diagnoses or treatments in Celiac disease: evidence of diagnostic delay. Scand J Gastroenterol.

[50] Shahbazkhani B, Sadeghi A, Malekzadeh R, Khatavi F, Etemadi M, Kalantri E (2015). Non-Celiac Gluten Sensitivity Has Narrowed the Spectrum of Irritable Bowel Syndrome: A Double-Blind Randomized Placebo-Controlled Trial. Nutrients.

